# Lactic Acid Bacteria–Yeast Consortia Enhance Nutritional Quality, Safety, and Volatilome of Fermented Chickpea Flour

**DOI:** 10.3390/foods15071239

**Published:** 2026-04-04

**Authors:** Solidea Amadei, Davide Gottardi, Marta Sindaco, Irene Gandolfi, Margherita D’Alessandro, Luisa Pellegrino, Mattia Di Nunzio, Lorenzo Siroli, Francesca Patrignani, Rosalba Lanciotti

**Affiliations:** 1 Department of Agricultural and Food Sciences, Campus of Food Science, Alma Mater Studiorum, University of Bologna, Piazza Goidanich 60, 47521 Cesena, Italy; solidea.amadei2@unibo.it (S.A.); irene.gandolfi4@unibo.it (I.G.); lorenzo.siroli2@unibo.it (L.S.); francesca.patrignani@unibo.it (F.P.); rosalba.lanciotti@unibo.it (R.L.); 2 Department of Food, Environmental and Nutritional Sciences (Defens), University of Milan, Via Celoria 2, 20133 Milan, Italy; marta.sindaco@unimi.it (M.S.); luisa.pellegrino@unimi.it (L.P.); mattia.dinunzio@unimi.it (M.D.N.); 3 Interdepartmental Centre for Agri-Food Industrial Research, Campus of Food Science, Alma Mater Studiorum, University of Bologna, Via Quinto Bucci 336, 47521 Cesena, Italy; margheri.dalessandr3@unibo.it

**Keywords:** fermented chickpea flour, protein content, carbohydrate content, volatilome, safety

## Abstract

Chickpea flour represents a valuable plant-based ingredient due to its high protein and fiber content; however, its application is limited by antinutritional factors and off-flavor compounds. Fermentation with LAB and yeasts, applied individually or in consortia, resulted in significant microbiological, nutritional, and aromatic changes. The fastest acidification (pH 3.9) and the most effective control of *Enterobacteriaceae* (<4 log CFU/g after 48 h) were observed in samples containing *Lactiplantibacillus plantarum* LP23, both as a monoculture and in combination with *Debaryomyces hansenii* Y15A. Peptide content significantly increased in all fermented samples compared to the control, with a synergistic effect in the co-culture *Yarrowia lipolytica* Y3 + *Lacticaseibacillus paracasei* L (around 230%). A pronounced reduction in raffinose-family oligosaccharides was observed, especially in the consortia *Y. lipolytica* Y3 + *Lcb. paracasei* L and *D. hansenii* Y15A + *Lacp. plantarum* LP23 (0.11–0.16 mmol/100 g). Samples with lower total volatile levels showed higher olfactory acceptability due to a marked reduction in aldehydes (up to 70–95% vs. control), and a balanced accumulation of alcohols, esters, ketones, and organic acids. Overall, LAB–yeast consortia effectively enhanced the nutritional quality, safety, and sensory properties of chickpea flour, supporting its use as a functional ingredient in plant-based foods.

## 1. Introduction

Chickpea (*Cicer arietinum* L.) is a legume belonging to the family *Fabaceae* and is the second most widely produced pulse worldwide [1]. As with other legumes, chickpeas are also a source of protein, fiber, vitamins and minerals. Due to their high protein content, legumes have recently been explored as an alternative source of proteins to meat, one that can be isolated and reintroduced into food formulations. However, protein extraction generates several by-products that, despite retaining nutritional and functional values, often remain underutilized. For this reason, employing legumes as whole ingredients is generally preferred, as it allows for the full exploitation of their nutritional potential. In fact, beyond their high protein content, regular consumption of legumes is also strongly associated with a reduced incidence of chronic conditions such as cardiovascular diseases, obesity, hypercholesterolemia, and type 2 diabetes [2]. Consequently, legume-derived flours are increasingly being incorporated into various food formulations (such as baked goods, dairy alternatives, beverages, and snacks) to enhance their nutritional value, particularly by boosting protein and dietary fiber contents [3]. However, like many other legumes, chickpeas naturally exhibit several drawbacks related to their intrinsic composition.

In fact, they have a lower content of essential amino acids (i.e., lysine and sulfur-containing amino acids) compared to animal-derived foods [4]. Moreover, legumes contain antinutritional factors, including trypsin and chymotrypsin inhibitors, phytic acid, saponins and tannins, which may limit their nutritional potential by impairing protein digestibility and reducing the bioavailability of essential minerals such as iron, zinc, calcium, and magnesium [5,6]. Finally, this legume contains undesired compounds that can cause discomfort (digestive issues, gas production and flatulence) in humans, such as raffinose-family oligosaccharides (RFOs) [7,8], or decrease acceptability, such as undesirable volatile compounds (e.g., aldehydes) [9]. Although some of these aspects can be partially mitigated through technological approaches (e.g., soaking, germination, or cooking), the most effective methods often rely on biotechnological processes [10,11].

Fermentation has been applied by humans in food production for centuries, as it can enhance the nutritional, organoleptic, technological, safety, and functional attributes of the final product [12]. Research focusing on chickpea fermentation has predominantly explored the use of several lactic acid bacteria (LAB) and yeast strains [13,14,15]. Studies have shown that fermented chickpea flour exhibits a marked reduction in antinutritional (e.g., phytic acid) and undesirable compounds (e.g., RFOs, aldehydes), and a substantial improvement in key functional properties, such as antioxidant activity, protein functionality, and technological properties (e.g., improved oil absorption capacity, foaming capacity, and foam stability), compared to unfermented flour [10,15]. Biotechnological processes also enhance the quality and functionality of chickpea proteins by facilitating their hydrolysis into smaller polypeptides, free amino acids, and bioactive peptides. They can also enrich the essential amino acid profile and significantly boost the overall nutritional value of the fermented product [16]. These functional and structural modifications induced by fermentation depend on various factors, including the genus, species, and strain of the microorganisms used, their specific metabolic activities, the synergistic effects arising from co-inoculation, the nutrient composition of the substrate, and the conditions of the fermentation process [17]. In a previous work Amadei et al. [18] compared the effects of the fermentation with six LAB and five yeast strains on the nutritional quality (soluble protein and peptide content, sugar composition, phytate levels), volatile molecule profiles, and functional properties (antioxidant and prebiotic activity) of chickpea flour. The data obtained showed that the tested microorganisms, particularly *Lacticaseibacillus paracasei* L and *Saccharomyces cerevisiae* FB2, reduced high-molecular-weight proteins into peptides and reduced RFOs in a strain-dependent manner. In addition, yeast strains, particularly *Debaryomyces hansenii*, were the most effective in stimulating probiotic growth and phytate reduction, while LAB-fermented samples showed the highest antioxidant activity. The data obtained clearly demonstrated that tailored yeast strains can also enhance the nutritional and biofunctional properties of chickpea flour, making it a promising ingredient for high-protein functional foods. In this framework, the present study aims to investigate the effects of combining the best-performing LAB and yeast strains selected by Amadei et al. [18] to further improve the characteristics of chickpea flour beyond what can be achieved by using a single microorganism. In particular, the effects of the co-inoculation of LAB strains (*Lactiplantibacillus plantarum* LP23, *Lacticaseibacillus paracasei* L, and *Latilactobacillus sakei* M12A) with yeast strains (*Yarrowia lipolytica* Y3, and *Debaryomyces hansenii* Y15A) on hydrated chickpea flour’s nutritional value, volatile profile and functional properties were investigated and compared to the effects of the single strains. To reach this goal, in addition to the monitoring of microbial growth and pH changes during fermentation, the volatile molecule profiles and the general acceptability of the fresh fermented products were analyzed. In addition, the freeze-dried fermented samples were assessed for their peptide content and profile, free amino acid composition, total, damaged, and digestible starch content, and sugar composition (with a focus on glucose, sucrose, and raffinose-family oligosaccharides).

## 2. Materials and Methods

### 2.1. Microbial Cultivation

The five microbial strains used in this study were selected based on the results described in Amadei et al. [18] and comprise three lactic acid bacteria (LAB: *Lacticaseibacillus paracasei* L, *Lactiplantibacillus plantarum* LP23, and *Latilactobacillus sakei* M12A) and two yeasts (*Yarrowia lipolytica* Y3, and *Debaryomyces hansenii* Y15A). All the strains belong to the culture collection of the Department of Agricultural and Food Sciences, Alma Mater Studiorum University of Bologna (Cesena, Italy). Yeasts were cultured twice in Yeast extract Peptone Dextrose (YPD) broth (Oxoid, Basigstone, UK) and incubated with agitation at room temperature for 72 h. LAB were cultured twice in MRS broth (Oxoid, Basigstone, UK) and incubated for 24 h at 37 °C. Cultures were centrifuged at 5000 rpm for 10 min and the biomasses were resuspended in an equivalent volume of sterile saline solution (NaCl, 9 g/L) as inoculum for the chickpea flour matrix.

### 2.2. Preparation of the Hydrated Chickpea Flour

Chickpea flour was purchased from a local supermarket and produced by Molino Maraldi (Cesena, Italy). The matrix was prepared as described by Amadei et al. [18] by mixing chickpea flour with bottled water (1:2, *w*:*w*). The selected microorganisms were then inoculated either individually or in combination (one yeast strain and one LAB strain), as reported in Table 1.

Each condition was prepared in sterile flasks in two independent trials consisting of three biological replicates each (*n* = 6). The inoculum levels were approximately 5 and 6 log CFU/g for yeasts and LAB, respectively, in both single-strain and co-culture samples. Incubation was carried out for 48 h at 30 °C in sterile bottles. As a control, a sample was collected immediately after hydration and before microorganism inoculation (T0). A portion of this sample was immediately subjected to microbiological analyses and stored for volatilome analyses, while the remaining fraction was promptly frozen, freeze-dried, and used as the 0 h reference for the remaining chemical analyses. During fermentation, samples were collected at 24 and 48 h for microbiological analyses and pH measurements. At the end of the incubation period, fermented samples were subjected to microbiological analyses and odor evaluation. The remaining material was partially stored for volatilome analyses, while the rest was stored at −80 °C and subsequently lyophilized for 5 days using a Drywinner Heto freeze-dryer (Cambridge Biosystems, Cambridge, UK) to be used for the other analyses.

### 2.3. Microbiological Analyses and pH Measurements

Microbiological analyses performed after 0, 24, and 48 h of fermentation were based on plate counting using 1 g of each sample that was homogenized using a stomacher and then subjected to serial decimal dilution with sterile saline water (NaCl, 9 g/L). All the dilutions were plated on specific media, as performed by Amadei et al. [18], and then incubated in the following way: for *Bacillus cereus* agar base and YPD, 30 °C for 24–48 h, respectively; for VRBGA and MRS, 37 °C for 24–48, respectively. Colony-forming units (CFU) were enumerated and then used to calculate the microbial concentration. The presence/absence of *Listeria monocytogenes* and *Salmonella* spp. was determined in samples collected at the end of fermentation according to ISO 11290–1:2017 [19] and ISO 6579–1:2017 [20], respectively. For both pathogens, the enrichment procedure was carried out by homogenizing 25 g of sample with the appropriate enrichment medium (half-Fraser broth for *L. monocytogenes* and buffered peptone water and RVS broth for *Salmonella*) at a tenfold dilution. Following incubation under the conditions specified by the respective standards, each enriched sample was plated onto the corresponding selective agar media (Agar *Listeria* according to Ottaviani and Agosti (ALOA) and Listeria Selective agar base (LSO, Oxoid, Basigstone, UK) for *L. monocytogenes*, and Xylose Lysine Desoxycholate (XLD, Oxoid, Basigstone, UK) for *Salmonella*). The presence/absence of *Escherichia coli* O157:H7 and STEC was investigated with Thermo Scientific SureTect *Escherichia coli* O157:H7 and STEC Screening PCR Assay. The enrichment step was performed by mixing 25 g of sample with buffered peptone water at a tenfold dilution and incubating according to the protocol. After enrichment, samples were subjected to PCR amplification. pH measurements were performed on all the samples (*n* = 6). Measurements were taken at 24 h intervals during fermentation using a pH meter (SevenCompact, Mettler Toledo, Columbus, OH, USA).

### 2.4. Nitrogen Component

#### 2.4.1. Peptide Content

Peptides were extracted from chickpea flour samples collected before fermentation (control) and from all fermented samples at the end of the incubation period. Briefly, 0.1 g of each lyophilized sample was suspended in 1 mL of extraction buffer (50 mM NaH2PO4 + 100 mM NaCl, pH 7). The suspension was stirred for 60 min at 22 °C, then subjected to centrifugation at 14,000 *g* for 10 min at room temperature. The resultant upper layer was collected and stored at −18 °C until use. The spectrophotometric analysis of peptides in the extracts was conducted utilizing the Pierce Quantitative Colorimetric Peptide Assay, which incorporates a modified BCA reagent, in conjunction with a proprietary chelator. In the assay, Cu^+2^ is first reduced by the amide backbone of peptides under alkaline conditions, followed by the proprietary chelator coupling with the reduced copper to form a bright red complex with an absorption maximum of 480 nm. The resulting data were obtained by extracting each sample and subsequently analyzing it spectrophotometrically (*n* = 6). Results were compared with the concentration–response curve of a peptide digest reference standard, normalized to the total protein content of the extract [21] and expressed as % relative to the control.

#### 2.4.2. Peptide Fractionation

Peptide fractions were extracted from chickpea flour samples collected before fermentation (control) and from all fermented samples at the end of the incubation period. Briefly, 0.25 mg of each lyophilized sample was suspended in 2.5 mL of 0.1% trifluoroacetic acid (TFA) (*v*/*v*). The suspension was stirred for 60 min and then subjected to centrifugation at 14,000 *g* for 10 min at room temperature. The resultant upper layers were collected, filtered with 0.22 µm cellulose acetate membrane, and stored at −18 °C until use. Peptides were separated according to their hydrophobicity by RP-HPLC in a SIMMETRY300 C18 (5 μm) (4.6 mm × 250 mm) column (Waters, Milford, MA, USA) fitted on a LC-4000 Series HPLC system (Jasco Corporation, Tokyo, Japan), equipped with AS-4050 General Purpose HPLC Autosamplers and using an MD-4010 PDA detector (Jasco Corporation, Tokyo, Japan). Peptide fractionating was conducted at a flow rate of 0.8 mL/min with an isocratic gradient for 10 min of solvent A (0.1% TFA in deionized water) and a linear gradient from 1% of solvent A to 50% of solvent B (0.1% TFA in 100% acetonitrile) over 60 min monitored at 214 nm. Peptides were conventionally categorized into four distinct classifications based on their respective retention times (Rt): hydrophilic, with a Rt between 0 and 10 min; weakly hydrophobic, with a Rt between 10.1 and 26.6 min; moderately hydrophobic, with a Rt between 26.7 and 43.3 min; and strongly hydrophobic, with a Rt between 43.4 and 60 min. Chromatographic traces and quantitative evaluations were obtained using ChromNAV 2.0 software. The data were obtained by extracting each sample and subsequently performing chromatographic analyses (*n* = 6). Results are expressed as the relative percentage of the total chromatographic peak area of each sample, which was normalized to 100%.

#### 2.4.3. Free Amino Acids Profile

Free amino acid (FAA) content was determined by ion exchange chromatography using a Biochrom 30+ amino acid analyzer (Erreci, Milan, Italy), following the method described by Hogenboom et al. [22]. Freeze-dried samples from the control (non-fermented) and from all fermented samples collected at the end of the incubation period were deproteinized with 7.5% 5-sulfosalicylic acid to extract FAA. The obtained solution was diluted with 0.2 N lithium citrate buffer and filtered on 0.2 µm cellulose acetate filter (Millipore, Burlington, MA, USA) prior to injection. Amino acids were quantified using a multipoint calibration curve, and results were expressed as mg AA/100 g of freeze-dried sample. The data were obtained by extracting each sample and subsequently performing chromatographic analyses (*n* = 6).

### 2.5. Carbohydrate Component

#### 2.5.1. Damaged Starch

Damaged starch was measured according to the AACC 76-31.01 method. Analyses were performed on freeze-dried samples from the control (non-fermented) and from all fermented samples collected at the end of the incubation period (*n* = 6). Results are expressed as relative abundance of damaged starch (% *w*/*w*).

#### 2.5.2. Digestible Starch Content

The measurement of digestible starch (divided into rapidly, slowly, and total) was conducted in accordance with the protocols outlined in the Digestible and Resistant Starch Assay Kit (Megazyme International Ltd., Bray, Ireland). Analyses were performed on freeze-dried samples from the control (non-fermented) and from all fermented samples collected at the end of the incubation period (*n* = 6). Results are expressed as g/100 g.

#### 2.5.3. Sugar Concentrations

Sugar concentrations were determined in freeze dried samples from the control (non-fermented) and from fermented samples collected at the end of the incubation period using the Megazyme (Megazyme International Ireland Limited, Bray, Ireland) kit K-RAFGL 08/23 raffinose/sucrose/glucose assay, following the manufacturer’s instructions. After enzyme inactivation and sugar extraction, glucose, sucrose and raffinose-family oligosaccharides were determined by measuring the absorbance at 510 nm with the UV-visible spectrophotometer (UV-1800, Shimadzu, Kyoto, Japan) and expressed in mmol/100 g. Water and glucose (included in the kit) were used as negative and positive controls, respectively. Sugar content was calculated based on the assay protocol provided by the manufacturer. The analyses were performed for each sample collected from the two independent trials (*n* = 6).

### 2.6. Volatile Molecule Profile

Volatile molecule profiles were determined after 48 h of incubation. To assess the modifications induced by the fermentation process, a non-inoculated sample was used as a control. The results represent the mean of the six samples (*n* = 6) measured with Solid-Phase Microextraction coupled with a Gas Chromatography–Mass Spectrometry (SPME/GC-MS) technique. An internal standard (IS: 4-methyl-2-pentanol) was added to all samples at a final concentration of 2 mg/L and used for the quantification of individual molecules, expressed in mg/L equivalent to the IS, according to Amadei et al. [18].

### 2.7. Preliminary Odor Detection

At the end of the incubation period, the odor characteristics of the inoculated samples were assessed and compared with those of the control sample. The evaluation was carried out by five untrained participants, including staff members and research fellows from the Department of Agricultural and Food Sciences, Alma Mater Studiorum—University of Bologna. The aim of this assessment was to obtain a preliminary indication of the overall odor acceptability of the fermented samples and to verify whether they could be considered pleasant by potential consumers, rather than to perform a detailed sensory profiling. Participants were informed about the purpose of the study and provided written informed consent prior to participation. No personal data were collected, and responses were recorded anonymously as part of an internal, informal survey. The evaluation was limited exclusively to olfactory perception; no tasting or ingestion of the samples was involved. Given the preliminary and non-invasive nature of the assessment, formal ethical approval was not required.

### 2.8. Statistical Analysis

Statistical analysis was performed on all samples collected from two independent trials, each consisting of three biological replicates (*n* = 6). The significance of data between the means of all samples were evaluated using one-way ANOVA followed by Tukey’s HSD post hoc test at *p* < 0.05 performed with Statistica software (v. 8.0; StatSoft, Tulsa, OK, USA). The volatile molecule profiles were analyzed using principal component analysis (PCA) with Statistica software.

## 3. Results and Discussion

### 3.1. pH and Microbiological Characterization

The pH dynamics during fermentation showed clear differences among treatments (Table 2). All samples started from an initial pH of 6.39, which is comparable with the values obtained by Sáez et al. (2022) [15] and Chiacchio et al. (2025) [13], but acidification trends varied markedly depending on the applied inoculum. After 24 h, the most pronounced decrease was observed in samples inoculated with *Lacp. plantarum* LP23 (pH 3.98) and, to a slightly lesser extent, in those containing *Lacp. plantarum* L (4.24 ± 0.01) or its combinations with yeasts, such as Y3+LP23 (pH 4.01) and Y15A+LP23 (pH 4.00).

Conversely, the use of only yeasts (Y3, Y15A) showed limited acidification, with pH values remaining above 5.1–5.4 after 24 h. After 48 h, the acidification trend became even more pronounced in LAB-containing samples. LP23 alone reached the lowest pH (3.85), closely followed by Y3+LP23 (3.90) and Y15A+LP23 (3.89). Treatments containing strain *Lcb. paracasei* L also showed substantial acidification, particularly when co-cultured with Y15A (pH 3.93). By contrast, samples inoculated with only yeasts exhibited more modest reductions, with final pH values remaining above 4.4. Overall, samples that included LAB, especially *Lacp. plantarum* LP23, showed the most pronounced and rapid pH decrease, confirming the strong acidifying ability of the selected LAB strains. This trend was also observed in the studies of Chiacchio et al. (2025) [13] and De Pasquale et al. (2020) [23], who tested different strains of LAB on chickpea flour. These results are consistent with those reported by Amadei et al. [18], with the exception of the yeast samples, which in the present study exhibited markedly lower pH values. In fact, *Y. lipolytica* Y3 and *D. hansenii* Y15A reached pH 4.5 and 4.4 at 48 h compared with pH 5.8 and 5.9 in the previous work, due to the growth of naturally occurring LAB.

**Figure 1:**
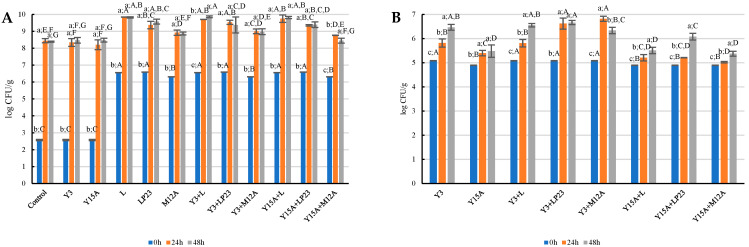
Lactic acid bacteria (**A**) and yeast (**B**) cell loads (log CFU/g) of chickpea flour inoculated with *Y. lipolytica* Y3 (Y3), *D. hansenii* Y15A (Y15A), *Lcb. paracasei* L (L), *Lacp. plantarum* LP23 (LP23), and *Latil. sakei* M12A (M12A), individually or in co-cultures, after 0, 24 and 48 h of incubation at 30 °C. Data for the control are not reported in panel B because yeast counts were below the limit of quantification (<1 log CFU/g). The results are the means of six replicates (*n* = 6) ± standard deviation. Significant differences (*p* < 0.05) within a sample during incubation time are indicated with lowercase letters, while significant differences among different samples at the same timepoint are indicated with capital letters.

Regarding the microbiological analyses, both yeasts and LAB increased their concentration during fermentation. In particular, all LAB strains reached their maximal concentrations after 24 h and maintained similar levels up to 48 h (Figure 1A). The best-performing strain was *Lcb. paracasei* L, followed by *Lacp. plantarum* LP23 and *Latil. sakei* M12A. These behaviors are consistent with the growth profiles previously reported by Amadei et al. [18] and similar to the results obtained by Chiacchio et al. [13], who tested only the use of LAB. LAB naturally present in the matrix were able to grow in both the control sample and those inoculated exclusively with yeast strains.

Regarding yeasts (Figure 1B), *Y. lipolytica* Y3 showed a progressive increase throughout the 48 h of incubation, reaching a final concentration of 6.4 log CFU/g. In contrast, *D. hansenii* Y15A grew more slowly, attaining only 5.4 log CFU/g after 48 h. Both yeasts grew slower compared to the data reported in Amadei et al. [18], particularly *D. hansenii*, which exhibited lower cell counts in the present study. In Amadei et al. [18], this strain was the fastest-growing among the yeasts within 48 h, although it was also the most affected by prolonged incubation: after 72 h its population declined, reaching approximately 6.5 log CFU/g. As mentioned above, this discrepancy may be attributed to the faster acidification kinetics observed in the present study compared to the previous one. This effect is likely associated with the growth of the spontaneous microbiota, including *Enterobacteriaceae* and lactic acid bacteria, which may have competed for fermentable substrates (e.g., sugars) and for the production of antimicrobial compounds, thereby influencing the overall fermentation dynamics. Nonetheless, the findings highlighted that *Y. lipolytica* Y3 appears to grow better in the matrix than *D. hansenii* Y15A.

When co-cultures were considered, *Y. lipolytica* Y3 exhibited enhanced growth, particularly in combination with *Lacp. plantarum* LP23 and *Latil. sakei* M12A. In these conditions, the yeast reached approximately 6.6–6.8 log CFU/g within 24 h, compared to 5.8 log CFU/g at 24 h in monoculture, and maintained this level until the end of the incubation period. Regarding *D. hansenii* Y15A, its growth was mainly stimulated in co-culture with *Lacp. plantarum* LP23, achieving a final concentration of 6.1 log CFU/g after 48 h. In control samples and in those inoculated with only LAB strains, yeasts were under the limit of quantification during all the incubation period.

Microbial interactions in co-culture can lead to positive, negative, or neutral effects on the partners involved [24]. In this context, the enhanced growth observed for *Y. lipolytica* Y3 in combination with LAB suggests a mutualistic or commensal relationship, where the presence of bacteria provides favorable conditions for yeast proliferation without an evident negative impact on the partner. Conversely, the limited stimulation observed for *D. hansenii* Y15A, restricted to its co-culture with *Lacp. plantarum* LP23, indicates that interactions can also be neutral or less beneficial, depending on the strain combination. These findings align with the broader ecological framework in which co-cultured microorganisms may support, inhibit, or have no measurable effect on each other, and highlight how strain-specific dynamics can strongly influence fermentation performance [25].

As reported by Amadei et al. [18] and De Pasquale et al. [23], this matrix can also support the growth of degradative and spoilage bacteria, particularly *Enterobacteriaceae*. The initial load was 2.48 log CFU/g across all samples (Figure S1), which is consistent with the results obtained by De Pasquale et al. [23]. After 24 h, *Enterobacteriaceae* increased in every condition; however, the rise was markedly lower in samples inoculated with LAB. In particular, *Lacp. plantarum* LP23, whether alone or combined with *D. hansenii* Y15A, limited *Enterobacteriaceae* growth to 5.30 and 5.70 log CFU/g, respectively, compared with values above 8.4–8.8 log CFU/g in yeast-only. This reduced growth could be related to the rapid pH drop of these samples (Table 2). After 48 h, *Enterobacteriaceae* decreased in all samples, a trend attributable again to pH reduction. The strongest inhibition occurred in LAB-inoculated matrices, where *Enterobacteriaceae* fell below detection limits (<4 log CFU/g) in several treatments, including LP23 alone and LP23 combined with Y15A. These results are consistent with the ability of LAB to produce organic acids, primarily lactic acid, that contribute substantially to acidification [26]. Conversely, yeasts mainly convert sugars into ethanol and CO_2_ or perform aerobic metabolism, exerting a more limited acidifying effect [27]. Interestingly, all the samples containing co-cultures of *Y. lipolytica* Y3 and LAB presented the highest values of *Enterobacteriaceae*, remaining above 5 log CFU/g. All these samples, however, exhibited similarly low pH values. This indicates that the inhibition of *Enterobacteriaceae* observed in the other samples was not solely attributable to acidification, but likely also to the presence of additional antimicrobial compounds that may have been metabolized or neutralized in the yeast-containing samples. Indeed, LAB, in addition to producing organic acids, can release bacteriocins, volatile antimicrobial molecules, and hydrogen peroxide [28,29]. The latter, in particular, can be readily degraded by yeast catalase and peroxidase enzymes, potentially reducing its inhibitory effect [30,31].

### 3.2. Protein Fraction

Chickpea flour contains approximately 21% of proteins that can undergo significant modifications upon fermentation [16]. These modifications may positively impact the digestibility and bioavailability of the fraction [31]. Focusing on the peptide content, it has been observed that the inoculation with several microbial strains both individually and in co-culture determined significant differences, as reported in Table 3.

Fermentation with individual strains resulted in a significant increase in peptide release, albeit to varying extents, contingent on the microorganism. In the samples that were fermented with yeasts, the one containing *Y. lipolytica* Y3 demonstrated an approximate increase of 152%. The sample that contained *D. hansenii* Y15A achieved a higher value of 204%, which is comparable to those observed in samples inoculated with LAB. Indeed, in LAB-fermented samples, *Lcb. paracasei* L showed the highest peptide content (216%), followed by those containing *Lacp. plantarum* LP23 (190%) and *Latil. sakei* M12A (179%). When co-inoculated cultures were examined, only one combination resulted in a peptide concentration that exceeded that of the corresponding monocultures. Specifically, the co-culture *Y. lipolytica* Y3 + *Lcb. paracasei* L reached 230%, indicating a clear synergistic proteolytic effect. All the other co-culture samples exhibited peptide levels that were not statistically different from those of the respective monocultures. The observed increases in peptide content following fermentation stem from the proteolytic activities of both LAB and yeasts, which release enzymes capable of hydrolyzing chickpea proteins into smaller peptides and free amino acids [10,16].

The increase in peptide content observed after fermentation can be attributed to the proteolytic activities of both LAB and yeasts. In LAB, protein hydrolysis is mainly initiated by cell-envelope proteinases, which generate peptides that are further degraded by intracellular peptidases; this proteolytic system is essential because LAB rely on exogenous peptides and amino acids due to their limited biosynthetic capacity [32,33]. Yeasts may also contribute through proteolytic enzymes. For instance, *Y. lipolytica* produces extracellular alkaline and acid proteases (XPR2 and AXP1), while *D. hansenii* possesses endoproteases such as protease A (PrA) and protease D (PrD), together with several aminopeptidases. These enzymes contribute to protein breakdown and the release of peptides and free amino acids during fermentation [34].

Importantly, peptides generated during fermentation may carry functional properties such as antioxidant, anti-inflammatory, antimicrobial, and antihypertensive activities, although their effects depend strongly on amino acid sequence and structure [35,36].

Peptide composition analysis revealed that, in all samples, hydrophobic peptides (the sum of strongly, moderately, and weakly hydrophobic peptides) represented the predominant fraction, accounting for approximately 65–75% of the total peptide content (Table 3).

Overall, the relative abundance of the strongly hydrophobic fraction increased in all the samples upon fermentation. This behavior was significant only for the samples containing *Y. lipolytica* Y3. For all the other samples, only a non-significant tendency toward higher values compared to the control was observed. From a functional standpoint, hydrophobic peptides are particularly relevant, as they have been associated with antioxidant and anti-inflammatory activities [37]. It is acknowledged that the peptide profile may undergo alteration during the process of digestion [38]. However, the modest increase in the strongly hydrophobic fraction observed in the fermented samples may possess the potential to enhance the biofunctional capacity in comparison with the unfermented control [39]. Differences were more pronounced in the hydrophilic peptide fraction. *Lacp. plantarum* LP23 and its co-cultures exhibited the highest relative abundance of hydrophilic peptides (approximately 39%, compared with 30% in the control). These variations likely reflect the distinct proteolytic specificities of the microbial strains involved.

From a functional perspective, hydrophilic peptides, or peptides rich in polar amino acids, are of notable interest: they contribute to key sensory attributes, particularly umami and savory notes, due to the presence of glutamic and aspartic acid residues [40]. Their high solubility also promotes bioaccessibility and digestion, facilitating their absorption and potential bioactivity [41]. Moreover, hydrophilic peptides (which contain lysine, arginine, histidine, serine, and threonine) readily participate in Maillard reactions with reducing sugars, influencing aroma development through the formation of specific and complex flavor notes [42]. Several studies have also associated hydrophilic peptides, including those derived from fermented legumes, with marked antioxidant and antihypertensive activities [43,44]. Taken together, the increased proportion of hydrophilic peptides in LP23-containing samples suggests an enhancement not only of nutritional and functional potential but also of the sensorial complexity of the fermented chickpea flour.

Regarding the free amino acids (FAAs), arginine and glutamic acid were the most abundant in the control sample, in agreement with the results of Zhao et al. [45] and consistent with the naturally high proportion of hydrophilic amino acids in chickpea flour. In all the fermented samples, the content of FAAs increased from 15 to 120% compared to the control (Table 4). In particular, chickpea flour inoculated with single strains showed the highest FAA content with *D. hansenii* Y15A (150.9 ± 4.1 mg AA/100 g), followed by the sample inoculated with *Latil. sakei* M12A (125.8 ± 5.0 mg AA/100 g) and *Lacp. plantarum* LP23 (108.5 ± 4.4 mg AA/100 g). The samples containing both yeasts and LAB presented an overall lower concentration of FAAs among the fermented samples, except for Y15A+LP23 and Y15A+M12A. This may be related to a lower release of FAAs or to a higher consumption and transformation in aroma compounds, since AAs are precursors of many molecules with sensory impact.

Fermentation markedly modified the FAA profile of chickpea flour. Essential branched-chain amino acids (BCAAs)—valine, isoleucine and leucine—showed a pronounced increase in most samples, with the highest values recorded in *D. hansenii* Y15A and *Latil. sakei* M12A (e.g., leucine increased from 1.3 ± 0.0 mg/100 g in the control to 13.9 ± 0.4 mg/100 g and 12.2 ± 0.1 mg/100 g, respectively). Hydrophobic amino acids (alanine, methionine, phenylalanine, proline and glycine) also increased significantly upon fermentation, reaching their maximum in Y15A (59.20 mg/100 g), followed by M12A (47.50 mg/100 g) and the consortium Y15A + M12A (45.20 mg/100 g). These amino acids are known contributors to antioxidant activity [46], and this trend corroborates previous findings in chickpea hydrolysates [47]. Among non-essential amino acids, strong increases were observed in aspartate and asparagine, particularly in Y15A and M12A.

Remarkably, arginine alone accounted for 52% of FAAs in the control sample, with 35.3 ± 1.4 mg/100 g. This content decreased substantially across fermentations, except for Y15A+LP23. Intermediate levels (roughly ranging between 16 and 30 mg/100 g) were recorded in LP23, Y3+LP23, Y15A+L and Y15A+M12A, while levels below 10 mg/100 g were recorded in the remaining samples. The decreased contents of arginine were accompanied by proportionally higher levels of citrulline and ornithine in most fermented samples. This pattern is consistent with the arginine deiminase (ADI) pathway, a pH-regulating mechanism in acid environments through which bacteria generate ammonia from arginine producing citrulline, which can be further converted into ornithine providing ATP [48,49]. Citrulline levels in fermented samples were all higher than in the control (0.1 mg/100 g) but fell into a rather limited range (1–6 mg/100 g). In contrast, ornithine levels varied more consistently with arginine depletion. A marked accumulation of ornithine occurred in Y15A (20.6 ± 0.6 mg/100 g) and M12A (21.2 ± 0.9 mg/100 g), far exceeding the control (0.2 mg/100 g). Trace levels of both citrulline and ornithine were recorded in Y15A+LP23, where arginine was not used. Citrulline is physiologically relevant, with recognized vasodilatory effects and roles in exercise recovery [50]. Importantly, both arginine depletion and ornithine accumulation occurred not only in LAB-inoculated samples but also in yeast-inoculated ones, although to a lesser extent. This pattern strongly suggests that the native chickpea microbiota contributed to arginine conversion, especially in yeast fermentations, where yeasts alone would not be expected to sustain such activity in the absence of a canonical ADI pathway. Notably, the consortia containing *D. hansenii* Y15A, especially Y15A + *Lacp. plantarum* LP23, showed distinctive accumulation of tyrosine compared with the control and most monocultures. Tyrosine decreased or remained stable in all other fermentations, indicating that its enrichment is a trait emerging specifically from Y15A-based interactions. Tyrosine serves as a precursor of catecholamine neurotransmitters (dopamine, norepinephrine, epinephrine), and its dietary availability has been linked to cognitive modulation under demanding conditions [51]. Fermentation also enhanced GABA levels across all treatments compared with the control, and a parallel decrease in glutamic acid accumulation was observed. LAB are recognized as efficient GABA producers, as converting glutamate into GABA, with proton consumption, is a common mechanism that contributes to intracellular pH homeostasis [48,52,53]. However, the highest GABA concentrations were found in yeast-inoculated samples (Y15A and Y3). Thus, in these fermentations the observed GABA enrichment likely reflects synergistic metabolic activity between inoculated microorganisms and the endogenous microflora.

Beyond nutritional enhancement, FAAs importantly shape sensory attributes. Aspartic and glutamic acids contribute to umami perception, while several amino acids participate in Maillard reactions with reducing sugars, influencing aroma development [45]. In the present study, Y15A + LP23 showed one of the highest glutamate levels (12.5 mg/100 g), potentially strengthening umami notes. Moreover, proline, responsible for sweet and caramel-like nuances, was markedly higher in Y15A (8.0 mg/100 g) than in the control and the other samples [54]. Altogether, these results demonstrate that fermentation not only improves the nutritional quality of chickpea flour but can also modulate its sensory profile through selective amino acid enrichment. At the same time, the biochemical transformations observed are the outcome of a complex interplay between the intrinsic properties of the chickpea matrix, its native microbiota, and the specific metabolic capabilities of the inoculated strains.

### 3.3. Carbohydrate Fraction

Beyond protein modification, a primary objective of this study was to modulate the content of carbohydrates, to have a more digestible product and to decrease the undesired components (RFOs). First, the total digestible starch content showed a slight increase in the inoculated samples compared with the control (28.41 ± 0.64 g/100 g). The highest value was observed in chickpea flour inoculated with *Y. lipolytica* Y3 (33.78 ± 0.30 g/100 g), followed by the Y3 + *Lcb. paracasei* L co-culture. For all other samples, only a tendency toward higher digestible starch levels was observed, without statistically significant differences. In all samples, the slowly digestible starch fraction represented the predominant component, with values ranging from 13.92 to 17.47 mg/100 g, similar to the results of Milán-Noris et al. [55], whereas the rapidly digestible fraction was less abundant, ranging from 2.12 to 3.35 mg/100 g. This distribution suggests that the samples are unlikely to induce a rapid increase in glycemic index, in line with the results for damaged starch content. However, no significant differences were observed among samples with respect to the control (Table 5).

Then, the content of damaged starch was determined. The values were significantly lower in the fermented samples compared to control (0.77%), with the lowest value (0.30%) estimated for the samples co-inoculated with *D. hansenii* Y15A and *Lacp. plantarum* LP23 (Table 5). This finding is consistent with a previous study demonstrating that microbial fermentation could contribute to a reduction in damaged starch content. This is due to the fact that microbial fermentation represents the second source of fermentation, and it is degraded to provide energy for microbial growth [56]. It should be noted, however, that damaged starch, when present, is structurally more accessible to digestive enzymes because it facilitates water penetration and enzymatic attack [57]. Therefore, while damaged starch is intrinsically more digestible, its reduction in the fermented samples reflects a shift toward a starch profile that is overall less rapidly digestible and thus nutritionally more favorable.

The concentrations of glucose, sucrose, and RFOs were also quantified before and after fermentation. As reported in Table 2, the initial D-glucose content was 1.88 ± 0.02 mmol/100 g, consistent with the values previously described by Amadei et al. [18] and Gangola et al. [58]. In agreement with that study, all strains tested were able to partially consume glucose during incubation, with the lowest residual concentration (1.53 ± 0.02 mmol/100 g) observed in samples inoculated with *Y. lipolytica* Y3 alone. Comparable reductions were also detected in the co-cultures Y3 + *Lcb. paracasei* L and Y3 + *Lacp. plantarum* LP23. In contrast, the sample inoculated with *D. hansenii* Y15A did not show the expected decrease in glucose; instead, a slight increase was observed. This discrepancy may be attributed to the shorter fermentation time applied in the present study (48 h vs. 72 h in Amadei et al. [18]), as well as to the lower growth performance of Y15A recorded here, which likely limited its glucose utilization capacity.

Sucrose content was significantly reduced in all inoculated samples compared with the control (8.15 ± 0.03 mmol/100 g), and in several cases, namely both yeast monocultures, LP23, M12A, Y15A+LP23, and Y15A+M12A, the residual sucrose fell below the limit of detection. The extent of sucrose depletion varied among treatments, independent of whether a single strain or a microbial consortium was applied. Notably, all samples containing *Lcb. paracasei* L, either alone or in co-culture, showed the smallest reduction in sucrose, maintaining comparatively higher levels. This trend aligns with the findings of Amadei et al. [18], where the same strain also exhibited limited ability to metabolize sucrose [59]. Both LAB and yeasts are known to possess diverse carbohydrate-hydrolyzing enzymes, enabling the breakdown of disaccharides into simpler molecules [60,61]. In particular, LAB possess transport systems and intern metabolic pathways able to hydrolyze sucrose [59,62], while sucrose hydrolysis in yeasts is usually extracellular or with periplasmatic invertase [63]. Additionally, endogenous enzymes and the native microbial community naturally present in chickpea flour may contribute to sucrose hydrolysis. Thus, the observed reductions likely reflect the combined action of inoculated strains together with the metabolic activities already present within the flour’s intrinsic microbiota. A significant decrease in RFO content was observed in all fermented samples compared with the control (1.39 ± 0.02 mmol/100 g), with the sole exception of the sample inoculated with *Lcb. paracasei* L, which showed no substantial reduction. The control value was consistent with previously reported data for chickpea flour [18,58]. The strongest RFO degradation occurred in the sample inoculated with the co-culture *Y. lipolytica* Y3 + *Lcb. paracasei* L (0.11 ± 0.03 mmol/100 g), followed by *Lacp. plantarum* LP23 (0.12 ± 0.02 mmol/100 g), *D. hansenii* Y15A (0.16 ± 0.00 mmol/100 g), Y15A+ L, and Y15A+LP23 (0.16 ± 0.03 mmol/100 g). Both bacteria and yeasts can possess α-galactosidase enzymes responsible for RFO hydrolysis. In yeasts, this activity is typically extracellular, whereas in LAB it is predominantly intracellular or cell-associated [18]. The enhanced degradation observed in certain co-cultures may therefore arise from complementary metabolic mechanisms between yeasts and LAB, further supported by the activity of the native microbiota naturally present in chickpea flour.

### 3.4. Volatile Molecule Profiles

**Figure 2:**
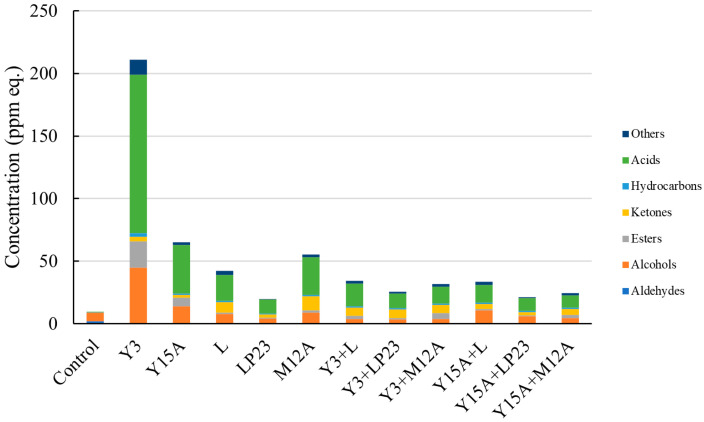
Volatile molecule concentration (ppm eq.) of chickpea flour not-inoculated (Control) and inoculated with yeast strains *Y. lipolytica* Y3 (Y3), *D. hansenii* Y15A (Y15A), *Lcb. paracasei* L (L), *Lacp. plantarum* LP23 (LP23), or *Latil. sakei* M12A (M12A), in single or in co-culture at the end of the incubation period. The results represent the mean of the six replicates (*n* = 6).

The analysis of the volatile molecule profiles with SPME/GC-MS allowed us to highlight the differences among the fermented and unfermented samples. Around 70 volatile molecules were detected and identified and are listed in Table S1. The control sample presented a total of 9.31 ppm eq., consisting of 74.7% of alcohols and 21.9% of aldehydes, which are the two dominant chemical classes in this sample. In particular, the most abundant volatile compounds were 1-hexanol, hexanal, and ethanol (46.0, 17.4, and 11.0%, respectively). Hydrocarbons, esters, and ketones were also present, representing 2.4, 0.4, 0.3, and 0.2% of the total relative abundance, respectively. These proportions are consistent with those reported by Khrisanapant et al. [64], who also observed that the high abundance of aldehydes in chickpea is associated with the activity of enzyme complexes such as lipoxygenases, isomerases, and other oxidative enzymes. Lipoxygenases catalyze the oxidation of polyunsaturated fatty acids, such as linoleic and α-linoleic acids, leading to the formation of C9 and C13 hydroperoxides, which are subsequently converted by hydroperoxide lyases into primary aldehydes, including hexanal and nonenals. These primary aldehydes can undergo further enzymatic or non-enzymatic transformations, such as oxidation and isomerization, resulting in the formation of additional volatile aldehydes such as pentanal, heptanal, 2-hexenal, octanal, 2-heptenal, and 2-octenal. Such compounds are typically associated with grass and beany notes, which are usually considered undesirable by consumers [16,65]. Moreover, the high abundance of alcohols in chickpea flour can be explained by the fact that it contains isozymes of alcohol dehydrogenases which catalyze the interconversion of aldehydes, acids, and alcohols, leading to the production of several alcohols, such as 1-hexanol, and acids, such as hexanoic acid [64]. Also 1-penten-3-ol and 1-octen-3-ol could be present in chickpea flour due to enzymatic pathways, and they are usually related to undesired, beany odor [3,64]. The fermentation process determined the accumulation of higher amounts of volatile compounds from 19.71 ppm eq. quantified in LP23 to 210.86 ppm eq. in Y3 (Figure 2).

To obtain a preliminary overview of the behavior of the samples, principal component analysis (PCA) was performed, where factor 1 explained 44.76% of the variance and factor 2 explained 22.76% of the variance (Figure S2).

Samples inoculated with LAB, co-inoculated with LAB and yeasts, as well as the sample inoculated with Y15A, clustered in the same region of the PCA plot, indicating similar volatile profiles. In contrast, the chickpea flour inoculated with Y3 was located in a distinct quadrant, clearly separated from the control sample. Although comparable yeast counts were observed in both single and co-culture fermentations, the chickpea flour used in this study was not sterilized and thus retained its native microbiota. During fermentation, this indigenous microflora may have interacted with the inoculated microorganisms in a manner dependent on the specific culture conditions. In co-culture systems, the activity of lactic acid bacteria likely promoted rapid acidification, which may have influenced both the native microbial community and, indirectly, the metabolic activity of yeasts. Such microbial interactions can affect the availability of key metabolic precursors and, consequently, the formation of volatile organic compounds (VOCs). Therefore, despite similar yeast cell counts, the metabolic behavior of strain Y3 may have been altered in the presence of other microorganisms, resulting in the lower VOC production observed in co-cultures. In particular, the results showed that, as expected, the control was mainly characterized by aldehydes, particularly acetaldehyde, pentanal, hexanal, 2-hexenal, (E)-, octanal, and nonanal, which are usually associated with beany flavor, and alcohols, such as 1-octen-3-ol and 1-heptanol, which are perceived as mushroom and vegetable odors [65]. The sample inoculated with *Y. lipolytica* Y3 showed an unique distinctive profile that was characterized by acids, such as acetic acid (vinegar odor, which could come from free amino acid degradation) and butanoic acid (buttery, rancid), and esters, particularly ethyl acetate (fruity, caramel aroma), butanoic acid, ethyl ester (sweet, buttery), acetic acid, butyl ester (sweet, fruity), and 1-butanol, 3-methyl-, and acetate (fruity, banana). Due to the polarizing characteristics of control and Y3, all the other fermented samples clustered together, making their description more complicated. However, these samples were characterized by a reduced content of aldehydes (70.77% reduction compared to control) and a sample-dependent content of alcohols.

**Figure 3:**
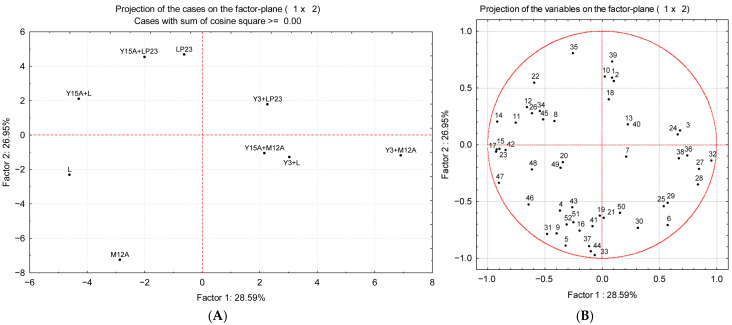
Score plot (**A**) and loading plot (**B**) obtained by PCA elaboration of the volatile organic compounds that characterize chickpea flour inoculated with *Lcb. paracasei* L (L), *Lacp. plantarum* LP23 (LP23), or *Latil. sakei* M12A (M12A), in single or in co-culture with *Y. lipolytica* Y3 (Y3) or *D. hansenii* Y15A (Y15A), at the end of the incubation period. **1**: Hexanal; **2**: 2-Heptenal, (E)-; **3**: 2-Octenal, (E)-; **4**: Benzaldehyde; **5**: Ethanol; **6**: 2-Butanol, (R)-; **7**: 1-Propanol; **8**: 1-Propanol, 2-methyl-; **9**: 1-Butanol; **10**: 1-Penten-3-ol; **11**: 1-Butanol, 3-methyl-; **12**: 3-Buten-1-ol, 3-methyl-; **13**: 2-Hexanol, 5-methyl-; **14**: 2-Buten-1-ol, 3-methyl-; **15**: 1-Hexanol; **16**: 1-Pentanol, 4-methyl-; **17**: 3-Hexen-1-ol, (Z)-; **18**: 4-Heptanol, 2,6-dimethyl-; **19**: 1,6-Octadien-3-ol, 3,7-dimethyl-; **20**: trans-3(10)-Caren-2-ol; **21**: 2-Heptanol, 5-ethyl-; **22**: 1-Nonanol; **23**: Benzyl alcohol; **24**: Phenylethyl Alcohol; **25**: Ethyl Acetate; **26**: Acetic acid; **27**: Butanoic acid, methyl ester; **28**: Butanoic acid, ethyl ester; **29**: 1-Butanol, 3-methyl-, acetate; **30**: Propanoic acid, 2-hydroxy-, ethyl ester, (S)-; **31**: Oxalic acid, cyclohexylmethyl tetradecyl ester; **32**: Acetone; **33**: 2-Butanone; **34**: 2-Heptanone; **35**: Acetoin; **36**: 3(2H)-Thiophenone, dihydro-2-methyl-; **37**: 2(3H)-Furanone, dihydro-5-pentyl-; **38**: D-Limonene; **39**: Heptane, 1,7-dibromo-; **40**: Cycloheptane; **41**: cis-2-Oxabicyclo[4.4.0]decane; **42**: Acetic acid; **43**: Propanoic acid; **44**: Butanoic acid; **45**: Hexanoic acid; **46**: Pentanoic acid, 4-methyl-; **47**: Hexanoic acid; **48**: Octanoic acid; **49**: Nonanoic acid; **50**: Phenol, 2-methoxy; **51**: Phenol; **52**: Heptaethylene glycol.

Indeed, based on a preliminary sensory evaluation, some differences were observed among the fermented samples. In particular, although not significant, a negative correlation (r = −0.70) was estimated between appreciation and total ppm eq. In fact, most of the samples that turned out to be the most interesting presented a total ppm eq. below 35 (LP23, Y3+L, Y3+LP23, Y3+M12A, Y15A+LP23). On the other hand, concentrations above this value were considered less appreciated, particularly samples Y3 and Y15A. To better understand the differences among the fermented samples that received positive scores, a PCA was performed only with them, where factor 1 explained 28.59% of the variance and factor 2 explained 26.95% of the variance (Figure 3A).

Samples fermented exclusively with LAB clustered on the left side of the PCA plot. In particular, samples inoculated with L and M12A were distributed in the lower-left quadrant, which was mainly characterized by propanoic (**43**), octanoic (**48**), hexanoic (**47**) and nonanoic acid (**49**). Conversely, LP23 was positioned in the upper-left quadrant, characterized by acetoin (**35**), as observed in Figure 3B. In the same area as LP23, the samples fermented with the co-culture of Y15A+L or Y15A+LP23 were also grouped, indicating that these combinations shared a volatile profile strongly influenced by LP23 activity. On the right side of the PCA plot, all the Y3 co-cultures, together with the sample Y15A+M12A, were clearly separated from the LAB-only fermentations. Overall, this distribution reflects the distinct metabolic contributions of yeasts and LAB to the accumulation of volatile compounds. For instance, all samples involving LP23 clustered in the upper portion of the PCA space, both left and right, confirming the strong and consistent impact of this strain on the product volatile profile.

It has already been demonstrated that in sourdough production, the combination of yeasts and LAB imparts a more complex and intense aroma compared to fermentation involving LAB alone [66,67]. This synergistic interaction enriches the volatile profile of the product, as yeasts mainly contribute to alcohols and esters, while LAB are responsible for the production of various organic acids [68]. Similarly, the findings of this study indicate that microbial inoculation, with both LAB and yeasts, enhanced the olfactory complexity of chickpea flour, likely due to the combined effect of diverse volatile compounds, their concentrations and thresholds. These molecules imparted distinctive aroma notes to the samples, which recall bakery, dairy and traditionally fermented products [18,54,69,70].

### 3.5. Safety Assessment

Chickpea flour provides a suitable environment for microbial growth, including potential foodborne pathogens, which were investigated in this study using multiple analytical approaches. Plate counts on selective media confirmed the absence of *Bacillus cereus*, *Listeria monocytogenes*, and *Salmonella* spp. Although *Enterobacteriaceae* were detected by plate counting, *Escherichia coli* was not isolated. qPCR analysis revealed the absence of *E. coli* O157:H7 and STEC in all six replicates of fermented chickpea flour, with the exception of samples inoculated with the strain L and with the co-culture Y15A+M12A, for which one out of six replicates tested positive. These results indicate that the pathogen, when present, occurred at very low levels, likely below the detection limit of conventional plate-counting methods (i.e., <1 CFU/100 g of sample).

The use of lactic acid bacteria is well-established for improving the safety of fermented food. In fact, these microorganisms produce various antibacterial compounds, including organic acids, which can inhibit the growth of foodborne pathogens. In particular, organic acids produced by LAB have been shown to inhibit the growth of *Listeria monocytogenes* and *Salmonella enterica* [54]. Recently, several yeast strains have also been investigated as biocontrol agents against pathogenic microorganisms [71,72]. Numerous studies have demonstrated that both lactic acid bacteria and yeasts are able to produce antimicrobial substances, including organic acids, ethanol, and hydrogen peroxide. For instance, LAB produce bacteriocins and phenyl-lactic acids that lower pH and directly inhibit pathogens such as *Listeria* and *Salmonella* in various food matrices [73]. Yeasts, on the other hand, have been shown to produce killer toxins, volatile organic compounds, and other inhibitory metabolites that exert antagonistic effects against both bacterial and fungal competitors in fermentation contexts [74]. Therefore, the co-inoculation of LAB and yeasts may have a synergistic effect, as yeasts stimulate the growth of LAB by providing essential metabolites such as vitamins, while LAB produce organic acids, which could be consumed by yeasts. In that way, the inoculated microorganisms could dominate against foodborne pathogens present in the food matrix [75]. Moreover, fermentation contributes to the creation of an unfavorable environment for the growth of pathogenic microorganisms due to the decrease in pH in the matrix. Specifically, pH values below 4.9 inhibit the growth of *Bacillus cereus*, and values below 4.6 are restrictive for *Clostridium botulinum*, below 4.5–4.8 for *Listeria monocytogenes*, below 4.3 for *E. coli*, and below 4.2 for *Salmonella* spp. [76]. At the end of the incubation period, all inoculated samples showed pH values of below 4.5, thus representing an environment unsuitable for the growth of these pathogens. Among them, samples inoculated with *Lacp. plantarum* LP23, either alone or in combination with yeast strains, exhibited the lowest pH values (below 3.9), indicating a particularly restrictive environment for pathogen development.

## 4. Conclusions

This study suggests that fermentation may represent a promising strategy to improve the nutritional, microbiological, and aromatic characteristics of chickpea flour, and that the use of selected LAB–yeast consortia may further contribute to these modifications compared to single-strain inoculation. Overall, the fermentation process was associated with marked biochemical changes, including an increase in peptide content and free amino acids, together with a reduction in undesirable carbohydrates, particularly raffinose-family oligosaccharides, which are responsible for gastrointestinal discomfort and represent a major technological limitation for legume-derived ingredients.

From a microbiological perspective, inoculated fermentations promoted rapid acidification and contributed to the control of *Enterobacteriaceae*, with the most effective performance observed in samples inoculated with *Lactiplantibacillus plantarum* LP23, either alone or in combination with *Debaryomyces hansenii* Y15A. Under these conditions, final pH values below 3.9 and *Enterobacteriaceae* levels below 4 log CFU/g after 48 h suggested the establishment of an environment restrictive for the growth of undesirable microorganisms. However, the results also indicate that the microbiological outcome of fermentation strongly depends on the microbial strains selected. While some inoculated fermentations (e.g., *Lpb. plantarum* LP23) reduced *Enterobacteriaceae* levels compared with spontaneous fermentation (control), other combinations resulted in similar or even higher levels (e.g., Y3+M12A). These findings highlight that the use of non-optimized microbial consortia may not ensure improved microbiological stability, emphasizing the importance of a tailored selection of starter cultures for chickpea flour fermentation. In addition, pathogen screening confirmed the absence of *Listeria monocytogenes* and *Salmonella* spp., while qPCR detection of *E. coli* O157:H7 and STEC indicated that, when present, contamination occurred only at very low levels.

Fermentation also strongly affected the volatilome of chickpea flour, reducing the abundance of aldehydes associated with beany/grassy off-flavors and promoting the accumulation of alcohols, esters, ketones and mild organic acids, thereby increasing the aromatic complexity of the matrix. Notably, samples characterized by moderate volatile concentration and a balanced distribution of aroma-active compounds showed higher olfactory acceptability. However, this odor assessment should be considered a preliminary evaluation, and further studies, including more comprehensive sensory analyses, would be required to confirm these observations. Taken together, these results suggest that the selection of specific LAB–yeast combinations may represent a promising biotechnological approach to obtain fermented chickpea flour with improved safety, enhanced nutritional value, reduced antinutritional/undesired components, and an improved aromatic profile. Further studies will be necessary to confirm these findings and to better evaluate their technological and sensory implications. This may support the future application of fermented chickpea flour as a functional and sustainable ingredient for the development of novel plant-based foods.

## Figures and Tables

**Table 1:** List of the chickpea flour samples obtained with single-strain inoculum or a combination of one yeast strain and one LAB.

Sample	Abbreviation
**Single strain**	
*Yarrowia lipolytica* Y3	Y3
*Debaryomyces hansenii* Y15A	Y15A
*Lacticaseibacillus paracasei* L	L
*Lactiplantibacillus plantarum* LP23	LP23
*Latilactobacillus sakei* M12A	M12A
**Co-inoculum**	
*Yarrowia lipolytica* Y3+*Lacticaseibacillus paracasei* L	Y3+L
*Yarrowia lipolytica* Y3+*Lactiplantibacillus plantarum* LP23	Y3+LP23
*Yarrowia lipolytica* Y3+*Latilactobacillus sakei* M12A	Y3+M12A
*Debaryomyces hansenii* Y15A+*Lacticaseibacillus paracasei* L	Y15A+L
*Debaryomyces hansenii* Y15A+*Lactiplantibacillus plantarum* LP23	Y15A+LP23
*Debaryomyces hansenii* Y15A+*Latilactobacillus sakei* M12A	Y15A+M12A

**Table 2:** pH values of the samples of chickpea inoculated with yeast (*Y. lipolytica* Y3 (Y3) and *D. hansenii* Y15A (Y15A)) or LAB (*Lcb. paracasei* L (L), *Lacp. plantarum* LP23 (LP23) and *Latil. sakei* M12A (M12A)) individually or in combination and in the not-inoculated chickpea flour (control). Values were registered immediately before incubation (0 h) and after 24 and 48 h of incubation. The results are the means of three samples from two independent trials (*n* = 6) ± standard deviation. Significant differences (*p* < 0.05) within the same sample over incubation time are indicated by lowercase letters, while significant differences among different samples at the same time point are indicated by uppercase letters. When letters are not reported, no statistically significant differences were detected.

	0 h	24 h	48 h
Control	6.39 ± 0.02 ^a^	5.23 ± 0.01 ^b;A^	4.44 ± 0.04 ^c;A^
Y3	6.39 ± 0.02 ^a^	5.14 ± 0.01 ^b;C^	4.45 ± 0.03 ^c;A^
Y15A	6.39 ± 0.02 ^a^	5.20 ± 0.01 ^b;B^	4.43 ± 0.02 ^c;A^
L	6.39 ± 0.02 ^a^	4.24 ± 0.01 ^b;H^	4.01 ± 0.01 ^c;D^
LP23	6.39 ± 0.02 ^a^	3.98 ± 0.01 ^b;J^	3.85 ± 0.01 ^c;F^
M12A	6.39 ± 0.02 ^a^	4.39 ± 0.01 ^b;F^	4.20 ± 0.03 ^c;C^
Y3+L	6.39 ± 0.02 ^a^	4.23 ± 0.01 ^b;H^	4.20 ± 0.01 ^b;C^
Y3+LP23	6.39 ± 0.02 ^a^	4.01 ± 0.01 ^b;I^	3.90 ± 0.01 ^c;E,F^
Y3+M12A	6.39 ± 0.02 ^a^	4.43 ± 0.01 ^b;E^	4.32 ± 0.02 ^c;B^
Y15A+L	6.39 ± 0.02 ^a^	4.52 ± 0.01 ^b;D^	3.93 ± 0.02 ^c;E^
Y15A+LP23	6.39 ± 0.02 ^a^	4.00 ± 0.01 ^b;I,J^	3.89 ± 0.01 ^c;E,F^
Y15A+M12A	6.39 ± 0.02 ^a^	4.30 ± 0.01 ^b;G^	4.16 ± 0.01 ^c;C^

**Table 3:** Peptide content (%) and peptide profile (%) of not-inoculated (Control) and inoculated chickpea flour with *Y. lipolytica* Y3 (Y3), *D. hansenii* Y15A (Y15A), *Lcb. paracasei* L (L), *Lacp. plantarum* LP23 (LP23), and *Latil. sakei* M12A (M12A) individually and in co-culture, at the end of the incubation period. Results are the average of six replicates (*n* = 6) and are expressed as mean ± standard deviation. Different letters mean significantly different (*p* < 0.05). When letters are not reported, no statistically significant differences were detected.

Samples	Peptides (%)	Peptide Profile (%)			
			Hydrophobic		
		Hydrophilic	Weakly	Moderately	Strongly
Control	100.00 ± 7.94 ^d^	30.19 ± 0.57 ^b^	22.02 ± 0.61	43.77 ± 0.16	4.03 ± 1.02 ^b^
Y3	152.80 ± 8.44 ^c^	34.28 ± 2.60 ^a,b^	19.09 ± 4.78	34.03 ± 6.50	12.61 ± 0.88 ^a^
Y15A	204.62 ± 6.38 ^a,b^	39.06 ± 4.66 ^a^	19.48 ± 3.77	31.88 ± 7.11	9.58 ± 1.31 ^a,b^
L	216.68 ± 0.56 ^a,b^	34.38 ± 1.37 ^a,b^	23.30 ± 1.25	32.31 ± 1.32	10.01 ± 1.44 ^a,b^
LP23	190.39 ± 0.98 ^a,b,c^	39.08 ± 1.37 ^a^	18.66 ± 0.34	33.30 ± 2.19	8.95 ± 1.16 ^a,b^
M12A	179.69 ± 3.55 ^b,c^	40.04 ± 0.09 ^a^	21.04 ± 0.21	32.02 ± 2.20	6.90 ± 2.09 ^a,b^
Y3+L	230.56 ± 8.48 ^a^	37.88 ± 1.08 ^a^	20.92 ± 0.43	34.22 ± 3.64	6.98 ± 2.13 ^a,b^
Y3+LP23	212.88 ± 3.77 ^a,b^	39.84 ± 1.03 ^a^	17.89 ± 0.87	36.19 ± 4.09	6.08 ± 2.20 ^a,b^
Y3+M12A	176.41 ± 0.17 ^b,c^	39.83 ± 0.68 ^a^	21.19 ± 1.08	32.43 ± 2.86	6.55 ± 2.45 ^a,b^
Y15A+L	174.52 ± 10.25 ^b,c^	36.60 ± 1.08 ^a,b^	20.95 ± 0.45	36.42 ± 1.65	6.02 ± 2.28 ^a,b^
Y15A+LP23	170.10 ± 6.43 ^b,c^	39.67 ± 0.31 ^a^	17.78 ± 0.30	36.23 ± 1.79	6.31 ± 2.41 ^a,b^
Y15A+M12A	201.78 ± 9.43 ^b,c^	39.57 ± 0.35 ^a^	22.13 ± 1.14	33.12 ± 2.10	5.18 ± 0.61 ^b^

**Table 4:** Free amino acids profile (mg AA/100 g of freeze-dried sample) of not-inoculated (Control) and inoculated chickpea flour with *Y. lipolytica* Y3 (Y3), *D. hansenii* Y15A (Y15A), *Lcb. paracasei* L (L), *Lacp. plantarum* LP23 (LP23), and *Latil. sakei* M12A (M12A) individually and in co-culture, at the end of the incubation period. Results are the average of six replicates (*n* = 6) ± standard deviation. Different letters in a row indicate significantly different data (*p* < 0.05).

	Control	Y3	Y15A	L	LP23	M12A	Y3+L	Y3+LP23	Y3+M12A	Y15A+L	Y15A+LP23	Y15A+M12A
Asp	0.4 ± 0.0 ^i^	2.2 ± 0.2 ^g,h^	8.0 ± 0.2 ^a^	3.7 ± 0.2 ^d,e^	3.9 ± 0.2 ^d^	6.5 ± 0.2 ^b^	3.2 ± 0.1 ^e,f^	2.1 ± 0.1 ^h^	4.9 ± 0.2 ^c^	3.4 ± 0.1 ^d,e^	2.7 ± 0.1 ^f,g^	6.9 ± 0.3 ^b^
Thr	0.9 ± 0.0 ^h^	4.4 ± 0.1 ^b^	7.6 ± 0.1 ^a^	3.2 ± 0.1 ^c^	3.0 ± 0.0 ^c,d^	1.7 ± 0.0 ^f^	2.8 ± 0.0 ^d^	2.2 ± 0.0 ^e^	1.2 ± 0.0 ^g^	2.4 ± 0.1 ^e^	1.9 ± 0.1 ^f^	1.4 ± 0.1 ^g^
Ser	1.0 ± 0.0 ^c^	1.3 ± 0.1 ^b^	3.2 ± 0.0 ^a^	1.3 ± 0.1 ^b^	0.3 ± 0.1 ^f^	0.8 ± 0.0 ^d^	1.2 ± 0.0 ^b^	0.4 ± 0.0 ^f^	0.6 ± 0.0 ^e^	0.8 ± 0.0 ^d^	0.3 ± 0.1 ^f^	0.7 ± 0.0 ^d,e^
Asn	2.2 ± 0.1 ^h^	2.3 ± 0.0 ^g,h^	5.9 ± 0.1 ^a^	3.8 ± 0.1 ^d^	4.9 ± 0.1 ^b^	2.6 ± 0.1 ^f,g^	3.1 ± 0.2 ^e^	4.4 ± 0.2 ^c^	1.6 ± 0.1 ^i^	2.8 ± 0.1 ^e,f^	4.4 ± 0.1 ^c^	2.4 ± 0.1 ^g,h^
Glu	10.0 ± 0.4 ^b^	1.4 ± 0.1 ^f^	3.9 ± 0.1 ^d^	2.4 ± 0.2 ^e^	10.4 ± 0.5 ^b^	2.4 ± 0.2 ^e^	2.4 ± 0.1 ^e^	4.1 ± 0.2 ^d^	3.9 ± 0.2 ^d^	5.6 ± 0.2 ^c^	12.5 ± 0.7 ^a^	5.4 ± 0.4 ^c^
Gln	0.0 ± 0.0 ^e^	0.0 ± 0.0 ^e^	0.0 ± 0.0 ^e^	0.0 ± 0.0 ^e^	1.5 ± 0.1 ^a^	0.4 ± 0.1 ^d^	0.3 ± 0.0 ^d^	0.7 ± 0.0 ^c^	0.7 ± 0.0 ^c^	0.0 ± 0.0 ^e^	0.0 ± 0.0 ^e^	0.9 ± 0.1 ^b^
Gly	1.5 ± 0.1 ^h^	4.4 ± 0.2 ^b^	5.8 ± 0.1 ^a^	3.7 ± 0.2 ^c,d^	3.1 ± 0.1 ^e,f^	4.6 ± 0.2 ^b^	2.7 ± 0.1 ^e,f,g^	2.4 ± 0.1 ^g^	3.2 ± 0.2 ^d,e^	3.0 ± 0.1 ^e,f^	2.6 ± 0.2 ^f,g^	4.2 ± 0.3 ^b,c^
Ala	0.3 ± 0.0 ^h^	8.9 ± 0.3 ^a^	7.1 ± 0.1 ^b^	2.9 ± 0.1 ^e^	1.3 ± 0.0 ^g^	5.1 ± 0.2 ^c^	2.6 ± 0.1 ^e^	2.0 ± 0.1 ^f^	3.6 ± 0.2 ^d^	2.6 ± 0.1 ^e^	1.4 ± 0.1 ^g^	3.9 ± 0.3 ^d^
Cit	0.1 ± 0.1 ^i^	6.1 ± 0.2 ^a^	4.3 ± 0.2 ^d,e,f^	5.1 ± 0.3 ^b^	4.4 ± 0.3 ^c,d,e^	4.1 ± 0.2 ^e,f^	3.7 ± 0.1 ^f^	2.1 ± 0.1 ^g^	4.9 ± 0.3 ^b,c,d^	5.0 ± 0.2 ^b,c^	1.0 ± 0.1 ^h^	3.9 ± 0.3 ^e,f^
Val	0.6 ± 0.1 ^h^	5.1 ± 0.3 ^b^	7.6 ± 0.1 ^a^	2.4 ± 0.1 ^e^	2.3 ± 0.1 ^e,f^	4.9 ± 0.1 ^b^	1.9 ± 0.0 ^f,g^	1.7 ± 0.1 ^g^	3.1 ± 0.1 ^b,c,d^	1.6 ± 0.1 ^b,c^	1.6 ± 0.1 ^h^	4.3 ± 0.3 ^e,f^
Met	0.4 ± 0.0 ^g^	2.5 ± 0.1 ^c^	3.4 ± 0.1 ^a^	1.7 ± 0.1 ^e^	1.7 ± 0.1 ^e^	3.0 ± 0.2 ^b^	1.3 ± 0.0 ^f^	1.3 ± 0.0 ^f^	2.1 ± 0.1 ^d^	1.2 ± 0.1 ^f^	1.3 ± 0.1 ^f^	2.9 ± 0.3 ^b^
Ile	0.6 ± 0.0 ^f^	3.9 ± 0.2 ^b^	5.6 ± 0.2 ^a^	1.6 ± 0.2 ^e^	2.1 ± 0.2 ^d^	4.3 ± 0.2 ^b^	1.7 ± 0.1 ^d,e^	1.5 ± 0.1 ^e^	2.9 ± 0.1 ^c^	1.3 ± 0.1 ^e^	1.5 ± 0.1 ^e^	4.0 ± 0.3 ^b^
Leu	1.3 ± 0.0 ^h^	10.5 ± 0.4 ^c^	13.9 ± 0.4 ^a^	7.1 ± 0.5 ^d,e^	8.1 ± 0.4 ^d^	12.2 ± 0.1 ^b^	5.3 ± 0.4 ^f,g^	6.4 ± 0.3 ^e,f^	8.2 ± 0.2 ^d^	4.7 ± 0.2 ^g^	7.0 ± 0.4 ^d,e^	11.7 ± 0.9 ^b,c^
Tyr	0.8 ± 0.1 ^c^	0.0 ± 0.0 ^d^	0.0 ± 0.0 ^d^	0.0 ± 0.0 ^d^	0.6 ± 0.0 ^c^	0.0 ± 0.0 ^d^	0.0 ± 0.0 ^d^	0.6 ± 0.0 ^c^	0.7 ± 0.1 ^c^	0.0 ± 0.0 ^d^	1.3 ± 0.1 ^b^	1.6 ± 0.2 ^a^
Phe	1.0 ± 0.1 ^f^	7.0 ± 0.3 ^b^	7.8 ± 0.2 ^a,b^	4.2 ± 0.2 ^d,e^	4.4 ± 0.2 ^d^	7.7 ± 0.4 ^a,b^	3.4 ± 0.2 ^e^	4.1 ± 0.2 ^d,e^	5.7 ± 0.2 ^c^	3.4 ± 0.2 ^e^	4.4 ± 0.2 ^d^	8.0 ± 0.7 ^a^
Gaba	4.3 ± 0.3 ^f^	29.1 ± 1.5 ^a^	29.4 ± 1.4 ^a^	22.5 ± 1.5 ^b^	13.7 ± 0.8 ^d,e^	26.5 ± 1.4 ^a^	17.5 ± 1.0 ^c^	16.9 ± 1.0 ^c,d^	18.6 ± 0.8 ^c^	18.7 ± 0.8 ^c^	12.2 ± 0.7 ^e^	23.0 ± 1.7 ^b^
Orn	0.2 ± 0.0 ^g^	6.0 ± 0.2 ^d^	20.6 ± 0.6 ^a^	17.9 ± 0.9 ^b^	9.6 ± 0.4 ^c^	21.2 ± 0.9 ^a^	16.4 ± 1.0 ^b^	3.2 ± 1.0 ^e,f^	11.0 ± 0.4 ^c^	6.8 ± 0.3 ^d^	1.6 ± 0.1 ^f,g^	3.8 ± 0.3 ^e^
Lys	2.3 ± 0.2 ^f^	2.6 ± 0.1 ^f^	5.9 ± 0.2 ^a^	0.3 ± 0.0 ^h^	5.1 ± 0.2 ^b^	3.3 ± 0.2 ^d,e^	1.7 ± 0.0 ^g^	1.8 ± 0.0 ^g^	3.7 ± 0.2 ^c,d^	0.6 ± 0.0 ^h^	3.9 ± 0.2 ^c^	3.1 ± 0.3 ^e^
His	1.1 ± 0.0 ^e^	0.4 ± 0.0 ^f^	2.4 ± 0.1 ^b^	1.9 ± 0.0 ^c^	2.0 ± 0.1 ^c^	1.1 ± 0.0 ^e^	1.0 ± 0.0 ^e^	1.6 ± 0.0 ^d^	2.4 ± 0.1 ^b^	2.4 ± 0.1 ^b^	1.9 ± 0.1 ^c^	2.7 ± 0.2 ^a^
Arg	35.3 ± 1.4 ^a^	0.4 ± 0.1 ^f^	0.5 ± 0.1 ^f^	2.5 ± 0.0 ^f^	20.6 ± 1.0 ^c^	7.7 ± 0.5 ^e^	0.7 ± 0.0 ^f^	21.7 ± 0.0 ^c^	9.4 ± 0.5 ^e^	16.1 ± 0.7 ^d^	36.3 ± 1.9 ^a^	30.0 ± 2.6 ^b^
Pro	2.8 ± 0.1 ^e^	3.0 ± 0.1 ^e^	8.0 ± 0.2 ^a^	5.6 ± 0.4 ^b^	5.5 ± 0.1 ^b^	5.7 ± 0.1 ^b^	4.4 ± 0.3 ^c,d^	4.3 ± 0.3 ^d^	4.3 ± 0.1 ^d^	5.3 ± 0.7 ^b,c^	4.4 ± 0.3 ^c,d^	6.2 ± 0.4 ^b^
Total	67.1 ± 2.8 ^g^	101.5 ± 3.9 ^c,d^	150.9 ± 4.1 ^a^	93.8 ± 4.9 ^c,d,e^	108.5 ± 4.4 ^c^	125.8 ± 5.0 ^b^	77.3 ± 3.6 ^f,g^	85.5 ± 3.7 ^e,f^	96.7 ± 4.1 ^c,d,e^	87.7 ± 4.1 ^d,e,f^	104.2 ± 6.0 ^c^	131.0 ± 10.0 ^b^

**Table 5:** Digestible starch fractions (g/100 g) damaged starch (%) and sugars (mmol/100 g) of chickpea flour not-inoculated (Control) and inoculated with *Y. lipolytica* Y3 (Y3), *D. hansenii* Y15A (Y15A), *Lcb. paracasei* L (L), *Lacp. plantarum* LP23 (LP23), or *Latil. sakei* M12A (M12A), in single or in co-culture at the end of the incubation period. The results are the means of six replicates (*n* = 6) ± standard deviation. Different letters mean significant differences (*p* < 0.05). - indicates data below the detection limit. When letters are not reported, no statistically significant differences were detected within a fraction/type of sugar.

	Digestible Starch (g/100 g)			Damaged Starch (%)	Sugars (mmol/100 g)		
Sample	Rapid	Slow	Total		D-Glucose (mmol/100 g)	Sucrose (mmol/100 g)	RFOs (mmol/100 g)
Control	2.1 ± 0.8	14.3 ± 0.3	28.4 ± 0.6 ^c^	0.8 ± 0.1 ^a^	1.9 ± 0.0 ^b^	8.1 ± 0.0 ^a^	1.4 ± 0.0 ^b^
Y3	3.3 ± 1.1	17.4 ± 1.5	33.8 ± 0.3 ^a^	0.5 ± 0.0 ^b^	1.5 ± 0.0 ^e^	-	0.8 ± 0.0 ^d^
Y15A	2.5 ± 0.8	16.2 ± 0.0	29.1 ± 0.6 ^b,c^	0.4 ± 0.1 ^b^	2.8 ± 0.0 ^a^	-	0.2 ± 0.0 ^g^
L	2.6 ± 0.7	17.5 ± 0.2	31.7 ± 0.4 ^a,b,c^	0.4 ± 0.0 ^b^	1.5 ± 0.0 ^e^	1.8 ± 0.0 ^c^	1.6 ± 0.1 ^a^
LP23	2.9 ± 0.2	14.8 ± 1.4	31.0 ± 0.1 ^a,b,c^	0.3 ± 0.0 ^b^	1.6 ± 0.0 ^e^	-	0.1 ± 0.0 ^g^
M12A	2.7 ± 0.2	16.0± 0.4	30.0 ± 1.7 ^b,c^	0.3 ± 0.0 ^b^	1.5 ± 0.0 ^e^	-	0.4 ± 0.0 ^f^
Y3+L	3.0 ± 0.7	17.2 ± 0.3	32.4 ± 1.0 ^a,b^	0.4 ± 0.0 ^b^	1.5 ± 0.0 ^e^	0.8 ± 0.0 ^d^	0.1 ± 0.0 ^g^
Y3+LP23	2.7 ± 0.1	13.9 ± 4.2	31.7 ± 0.4 ^a,b,c^	0.3 ± 0.0 ^b^	1.5 ± 0.0 ^e^	0.0 ± 0.0 ^f^	0.4 ± 0.0 ^f^
Y3+M12A	2.9 ± 0.9	16.8 ± 1.5	31.6 ± 1.0 ^a,b,c^	0.4 ± 0.0 ^b^	1.5 ± 0.0 ^e^	0.1 ± 0.0 ^e^	0.6 ± 0.0 ^e^
Y15A+L	2.4 ± 0.8	16.9 ± 1.7	30.9 ± 0.9 ^a,b,c^	0.4 ± 0.0 ^b^	1.8 ± 0.0 ^c^	2.6 ± 0.0 ^b^	0.2 ± 0.0 ^g^
Y15A+LP23	2.5 ± 1.0	16.4 ± 2.0	30.3 ± 1.0 ^a,b,c^	0.3 ± 0.0 ^b^	1.6 ± 0.0 ^d^	-	0.2 ± 0.0 ^g^
Y15A+M12A	2.3 ± 1.0	15.9 ± 1.3	30.0 ± 1.4 ^b,c^	0.4 ± 0.0 ^b^	1.8 ± 0.0 ^c^	-	1 ± 0.0 ^c^

## Data Availability

The datasets supporting the conclusions of this article are available in the Zenodo repository, at https://zenodo.org/records/18492193 (accessed on 10 March 2026).

## Supplementary Materials

The following supporting information can be downloaded at https://www.mdpi.com/article/10.3390/foods15071239/s1, Figure S1: Cell loads (log CFU/g) of *Enterobacteriaceae* in the samples of chickpea flour inoculated with yeast (*Y. lipolytica* Y3 (Y3) and *D. hansenii* Y15A (Y15A)) or LAB (*L. paracasei* L (L), *L. plantarum* LP23 (LP23) and *L. sakei* M12A (M12A)) individually or in combination and in the not-inoculated chickpea flour (control). Cell loads were determined immediately before incubation (0 h) and after 24 and 48 h incubation at 30 °C. The results are the means of six replicates (*n* = 6) ± standard deviation. Significant differences (*p* < 0.05) within a sample during incubation time are indicated with lowercase letters, while significant differences among different samples at the same timepoint are indicated with capital letters; Table S1: Volatile organic components detected by SPME/GC-MS in chickpea flour not-inoculated (Control) and inoculated with *Y. lipolytica* Y3 (Y3), *D. hansenii* Y15A (Y15A), *Lcb. paracasei* L (L), *Lacp. plantarum* LP23 (LP23), or *Latil. sakei* M12A (M12A), in single or in co-culture at the end of the incubation period, and expressed as mg/L equivalent. Results are the average of six replicates (*n* = 6); Figure S2: Score plot obtained by PCA elaboration of the volatile organic compounds that characterize chickpea flour not-inoculated (Control), and inoculated with *Y. lipolytica* Y3 (Y3), *D. hansenii* Y15A (Y15A), *Lcb. paracasei* L (L), *Lacp. plantarum* LP23 (LP23), or *Latil. sakei* M12A (M12A), in single or in co-culture at the end of the incubation period.
