# Supplementary material for: Lactic Acid Bacteria–Yeast Consortia Enhance Nutritional Quality, Safety, and Volatilome of Fermented Chickpea Flour
Source: Foods. 2026 Apr 4;15(7):1239. doi: 10.3390/foods15071239 (PMC13073144; doi:10.3390/foods15071239)
Supplement: Supplementary file 1 [file foods-15-01239-s001.zip › foods-4167946-supplementary.pdf]

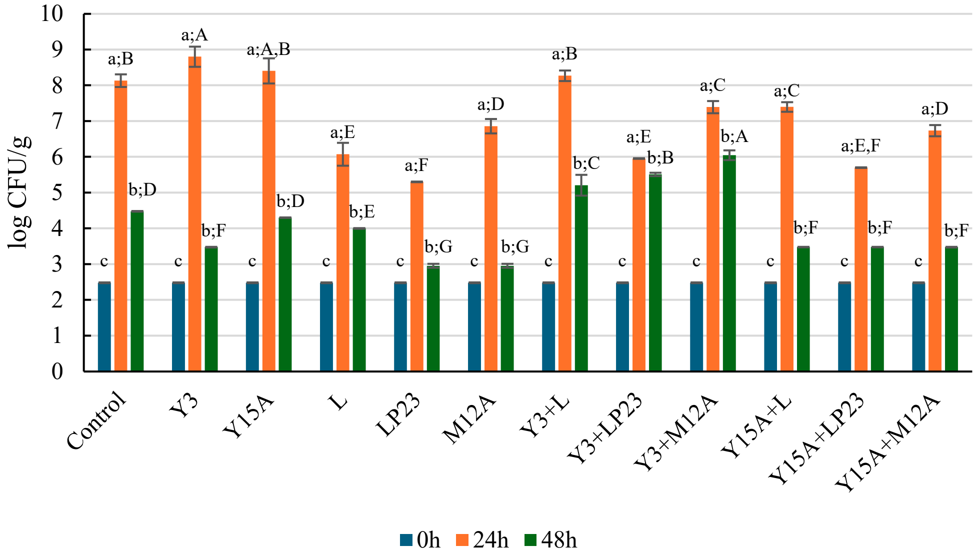

Figure S1. Cell loads (log CFU/g) of *Enterobacteriaceae* in the samples of chickpea flour inoculated with yeast (*Y. lipolytica* Y3 (Y3) and *D. hansenii* Y15A (Y15A)) or LAB (*L. paracasei* L (L), *L. plantarum* LP23 (LP23) and *L. sakei* M12A (M12A)) individually or in combination and in the not-inoculated chickpea flour (control). Cell loads were determined immediately before incubation (0 h) and after 24 and 48 h incubation at 30 °C. The results are the means of six replicates ($n = 6$) ± standard deviation. Significant differences ($p < 0.05$) within a sample during incubation time are indicated with lowercase letters, while significant differences among different samples at the same timepoint are indicated with capital letters.

Table S1. Volatile organic components detected by SPME/GC-MS in chickpea flour not-inoculated (Control) and inoculated with *Y. lipolytica* Y3 (Y3), *D. hansenii* Y15A (Y15A), *L. paracasei* L (L), *L. plantarum* LP23 (LP23), or *L. sakei* M12A (M12A), in single or in co-culture at the end of the incubation period, and expressed as mg/L equivalent. Results are the average of six replicates (*n* = 6).

Volatile organic compound	Control	Y3	Y15A	L	LP23	M12A	Y3+L	Y3+LP23	Y3+M12A	Y15A+L	Y15A+LP23	Y15A+M12A
Acetaldehyde	0.09	-*	-	-	-	-	-	-	-	-	-	-
Butanal, 3-methyl-	0.07	-	-	-	-	-	-	-	-	-	-	-
Pentanal	0.06	-	-	-	-	-	-	-	-	-	-	-
Hexanal	1.58	0.45	0.09	0.18	0.27	0.16	0.04	0.48	0.28	0.19	0.52	0.24
Heptanal	0.04	-	-	-	-	-	-	-	-	-	-	-
2-Hexenal, (E)-	0.04	-	-	-	-	-	-	-	-	-	-	-
Octanal	0.02	-	-	-	-	-	-	-	-	-	-	-
2-Heptenal, (E)-	0.03	-	-	-	0.01	-	-	0.03	-	-	0.03	0.01
Nonanal	0.03	-	-	-	-	-	-	-	-	-	-	-
2-Octenal, (E)-	0.02	-	-	-	-	-	-	0.02	0.04	-	0.01	-
Benzaldehyde	0.02	0.17	0.19	0.08	0.04	0.09	0.03	0.05	0.04	0.07	0.03	0.10
**Total aldehydes**	2.00	0.63	0.28	0.26	0.32	0.24	0.07	0.58	0.36	0.26	0.58	0.35
Ethanol	1.03	26.16	8.18	2.82	0.95	4.04	2.01	1.44	2.16	2.69	0.90	2.00
2-Butanol, (R)-	-	2.21	0.43	-	-	0.10	0.07	-	0.11	-	-	0.06
1-Propanol	0.03	-	-	-	-	-	-	-	-	-	-	0.10
1-Propanol, 2-methyl-	-	-	-	-	-	-	-	-	-	0.04	-	-
1-Butanol	-	0.84	0.40	0.08	0.03	0.25	0.02	0.01	-	-	-	0.02
1-Penten-3-ol	0.14	0.10	-	0.05	0.05	0.02	0.06	0.03	0.08	0.10	0.10	0.05
1-Butanol, 3-methyl-	0.21	1.82	0.85	1.04	0.73	0.88	0.26	0.47	0.27	2.32	1.02	0.67
3-Buten-1-ol, 3-methyl-	0.53	-	-	0.80	0.41	-	0.46	-	-	1.23	0.49	-
2-Hexanol, 5-methyl-	-	-	-	-	-	-	-	0.01	-	-	-	-

2-Penten-1-ol, (Z)-	0.06	0.30	-	-	-	-	-	-	-	-	-	-
2-Buten-1-ol, 3-methyl-	-	-	0.13	0.14	0.11	0.10	0.08	0.05	0.04	0.17	0.12	0.06
1-Hexanol	4.30	10.45	3.29	2.30	1.50	2.86	0.56	0.66	0.42	3.65	2.48	1.03
1-Pentanol, 4-methyl-	-	-	0.05	0.08	-	0.07	0.05	0.04	-	-	-	0.06
3-Hexen-1-ol, (Z)-	0.08	0.18	0.06	0.06	0.04	0.07	0.01	-	-	0.08	0.06	0.03
1-Octen-3-ol	0.07	-	-	-	-	-	-	-	-	-	-	-
1-Heptanol	0.20	-	-	-	-	-	-	-	-	-	-	-
4-Heptanol, 2,6-dimethyl-	-	-	-	-	0.05	-	0.02	-	-	-	-	-
2-Hepten-1-ol, (E)-	0.02	0.31	0.02	-	-	-	-	-	-	-	-	-
1,6-Octadien-3-ol, 3,7-dimethyl-	0.03	0.08	0.03	0.03	-	0.03	0.03	0.03	0.02	0.03	0.02	0.03
1-Octanol	0.09	0.27	-	-	-	-	-	-	-	-	-	-
trans-3(10)-Caren-2-ol	0.02	-	-	0.06	-	-	-	0.02	-	-	-	-
2-Decen-1-ol	-	0.34	-	-	-	-	-	-	-	-	-	-
2-Octen-1-ol, (E)-	0.01	-	-	-	-	-	-	-	-	-	-	-
1-[2-Methyl-3-(methylthio)allyl]cyclohex-2-enol	0.10	-	-	-	-	-	-	-	-	-	-	-
2-Heptanol, 5-ethyl-	-	0.16	-	-	-	0.04	0.04	-	-	-	-	-
1-Nonanol	0.07	0.15	0.13	0.05	0.04	-	-	-	0.01	0.04	0.05	0.01
Benzyl alcohol	-	0.12	0.05	0.03	0.02	0.04	-	0.01	-	0.04	0.03	-
Phenylethyl Alcohol	-	0.55	0.12	0.08	0.03	0.06	0.13	0.15	0.15	0.08	0.13	0.10
**Total alcohols**	6.98	44.05	13.74	7.61	3.96	8.56	3.79	2.95	3.28	10.46	5.38	4.23
Ethyl Acetate	0.03	14.26	4.52	0.20	0.04	0.87	0.16	0.11	1.53	0.26	0.11	0.85
Acetic acid ethenyl ester	-	-	0.18	0.49	0.29	0.20	0.33	0.31	0.22	0.51	0.32	0.26
Butanoic acid, methyl ester	-	-	-	-	-	-	0.18	-	0.32	-	-	0.11
Butanoic acid, ethyl ester	-	5.65	1.58	0.11	-	0.45	1.47	0.23	2.33	0.07	0.01	0.86

Acetic acid, butyl ester	-	0.09	-	-	-	-	-	-	-	-	-	-
1-Butanol, 3-methyl-, acetate	-	0.14	-	-	-	0.05	-	-	0.09	-	-	0.05
Propanoic acid, 2-hydroxy-, ethyl ester, (S)-	-	0.17	0.11	0.04	-	0.06	0.05	0.03	0.07	0.05	-	0.05
Oxalic acid, cyclohexylmethyl tetradecyl ester	-	0.75	0.29	0.24	0.11	0.30	0.17	0.18	0.15	0.17	0.18	0.16
**Total esters**	0.03	21.06	6.67	1.09	0.44	1.91	2.36	0.86	4.71	1.07	0.61	2.33
Acetone	-	-	-	-	0.05	0.07	0.41	0.37	0.49	-	0.04	0.29
2-Butanone	-	3.46	1.89	6.80	0.67	10.36	4.90	3.63	4.81	1.90	0.34	3.48
2-Heptanone	-	-	-	0.03	-	-	-	-	-	0.05	0.05	0.04
Acetoin	-	0.13	0.25	1.52	2.14	0.82	0.96	2.66	1.06	2.06	2.25	1.10
3(2H)-Thiophenone, dihydro-2-methyl-	-	-	-	-	-	-	0.39	0.22	0.26	-	-	-
2(3H)-Furanone, dihydro-5-pentyl-	0.02	0.13	0.05	0.03	0.02	0.04	0.03	0.03	0.03	0.03	0.03	0.03
**Total ketones**	0.02	3.72	2.19	8.39	2.88	11.28	6.70	6.90	6.65	4.04	2.71	4.94
D-Limonene	0.03	-	-	-	-	-	-	-	0.05	-	-	-
Heptane, 1,7-dibromo-	-	-	-	-	0.17	-	-	0.36	0.20	0.32	0.33	0.13
Cycloheptane	-	-	-	-	-	-	-	0.02	-	-	-	-
cis-2-Oxabicyclo[4.4.0]decane	0.22	3.08	1.13	0.99	0.46	1.13	0.86	0.98	0.91	0.84	0.89	0.86
**Total hydrocarbons**	0.25	3.08	1.13	0.99	0.63	1.13	0.86	1.35	1.17	1.15	1.22	1.00
Acetic acid	-	33.10	14.35	10.10	8.61	10.14	7.56	7.46	6.62	10.71	9.09	5.05
Propanoic acid	-	0.30	-	0.05	0.02	0.14	-	-	-	0.06	-	0.17
Butanoic acid	-	87.83	24.01	9.80	2.07	18.91	10.41	3.65	6.62	2.62	0.34	4.28
Hexanoic acid	-	0.22	0.10	0.06	0.05	0.06	0.05	0.06	0.05	0.05	0.08	0.05
Pentanoic acid, 4-methyl-	-	3.45	0.01	0.03	0.02	0.07	-	0.01	-	0.01	0.02	0.01
Hexanoic acid	0.03	1.20	0.47	0.47	0.24	0.48	0.21	0.20	0.13	0.36	0.37	0.20
Octanoic acid	-	0.15	0.04	0.04	0.02	0.03	0.03	0.01	-	0.01	0.04	0.03

Nonanoic acid	-	0.04	0.05	0.07	0.05	0.07	0.05	0.09	0.03	0.04	0.05	0.04
**Total acids**	0.03	126.29	39.05	20.62	11.08	29.90	18.31	11.48	13.45	13.87	10.00	9.83
Phenol, 2-methoxy-	-	0.36	0.11	0.08	0.03	0.08	0.05	0.06	0.10	0.08	0.06	0.09
Phenol	-	11.19	1.91	3.15	0.36	2.32	2.16	1.45	1.98	2.54	0.79	1.67
Indole	-	0.33	0.02	-	-	-	-	-	-	-	-	-
Heptaethylene glycol	-	-	0.05	0.05	0.01	0.05	-	0.03	0.03	0.01	-	0.01
Octaethylene glycol monododecyl ether	-	0.16	-	-	-	-	-	-	-	-	-	-
**Total others**	-	12.04	2.08	3.29	0.40	2.45	2.21	1.54	2.11	2.64	0.85	1.78

*: below the detection limit

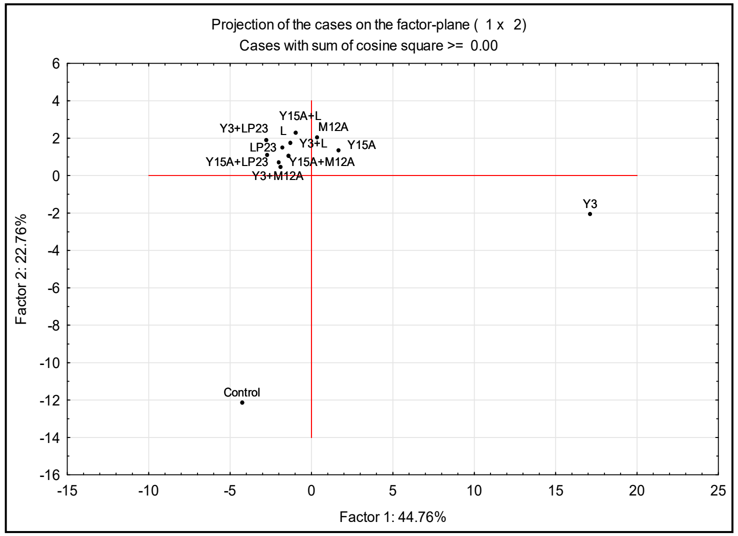

Figure S2. Score plot obtained by PCA elaboration of the volatile organic compounds that characterize chickpea flour not-inoculated (Control), and inoculated with *Y. lipolytica* Y3 (Y3), *D. hansenii* Y15A (Y15A), *L. paracasei* L (L), *L. plantarum* LP23 (LP23), or *L. sakei* M12A (M12A), in single or in co-culture at the end of the incubation period.
